# Parental Perception of Weight Status: Influence on Children’s Diet in the Gateshead Millennium Study

**DOI:** 10.1371/journal.pone.0144931

**Published:** 2016-02-17

**Authors:** Suzana Almoosawi, Angela R. Jones, Kathryn N. Parkinson, Mark S. Pearce, Heather Collins, Ashley J. Adamson

**Affiliations:** 1 Institute of Health & Society and Human Nutrition Research Centre, Newcastle University, Newcastle upon Tyne, NE2 4HH, United Kingdom; 2 Institute of Health and Society and Sir James Spence Institute of Child Health, Royal Victoria Infirmary, Newcastle upon Tyne, NE2 4HH, United Kingdom; 3 Faculty of Medical Sciences, Newcastle University, Newcastle upon Tyne, NE2 4HH, United Kingdom; National Institute of Agronomic Research, FRANCE

## Abstract

**Objective:**

Recognising overweight and obesity is critical to prompting action, and consequently preventing and treating obesity. The present study examined the association between parental perceptions of child weight status and child’s diet.

**Methods:**

Participants were members of the Gateshead Millennium Study. Parental perception of their child’s weight status was assessed using a questionnaire and compared against International Obesity Task Force cut-offs for childhood overweight and obesity when the children were aged 6–8 years old. Diet was assessed at age 6-8years old using the FAST (*Food Assessment in Schools Tool)* food diary method. The association between parental perception and dietary patterns as defined by Principal Components Analysis, was assessed using multivariate regression after adjustment for child’s gender, child’s weight status, maternal body mass index (BMI), maternal education and deprivation status.

**Results:**

Of the 361 parents who provided complete data on confounders and on their perception of their child’s weight status, 63 (17%) parents perceived their child as being of ‘normal’ weight or ‘overweight’ when they were actually ‘overweight’ or ‘obese’, respectively. After adjustment for confounders, parents who misperceived their child’s weight had children with a lower ‘healthy’ dietary pattern score compared to children whose parents correctly perceived their weight (β = -0.88; 95% CI: -1.7, -0.1; P-value = 0.028). This association was found despite higher consumption of reduced sugar carbonated drinks amongst children whose parents incorrectly perceived their weight status compared to children whose parents perceived their weight correctly (52.4% vs. 33.6%; P-value = 0.005).

**Conclusions:**

In conclusion, children whose parents did not correctly perceive their weight status scored lower on the ‘healthy’ dietary pattern. Further research is required to define parents’ diets based on their perception status and to examine if a child’s or parent’s diet mediates the association between parental perception and child weight.

## Introduction

Childhood overweight and obesity is a key public health priority [1]. It is increasingly recognised that parents’ perceptions of their child’s weight status is an important factor when planning public health interventions to reduce the prevalence of overweight and obesity [1].

Previously, we have demonstrated that parents’ ability to recognise overweight is limited [2,3]. These findings have been further supported by a recent review wherein 62.4% of parents were found to misperceive their child’s weight status [4]. Several factors have been shown to influence parental perception of child’s weight status including child’s sex, age, parental weight status, socioeconomic status and educational level [5]. For instance, parents are more likely to misperceive their child’s weight when their children are younger [6]. Likewise, parents who are overweight or obese more often perceive their child’s weight status as being normal when their child is actually overweight or obese [5]. Since parents who recognise their child’s weight status as a health problem are more likely to make changes to their children’s lifestyle [5,7], it is critical to understand if children whose parents correctly perceive their weight status have a healthier diet compared to parents who misperceive their child’s weight status. Parents are known to play an integral role in shaping children’s eating behaviour and to act as agents of change and role models promoting behavioural change in children [6]. Early intervention is critical to preventing or treating overweight and obesity as dietary patterns and habits often form in childhood and persist through adolescence into adulthood [8]. Moreover, several studies suggest that childhood obesity accounts for 25% of adult obesity and that overweight children have higher body mass index (BMI) trajectories in adulthood [4].

To date, limited research exists on the association between parental misperception of child’s weight status and diet. We aimed to examine the association between parental perceptions of their child’s weight status and child’s diet. We hypothesised that children whose parents misperceived their weight status are more likely to have an unhealthy dietary pattern.

## Methods

### Ethical Statement

Ethical approval was gained from Gateshead and South Tyneside Local Research Ethics Committee for baseline data collection and follow-up data collection at ages 6–8 years. Parents or main guardians gave informed written consent and children provided their assent to participation.

### Cohort and data collection

Participants were children and parents from the longitudinal Gateshead Millennium Study (GMS) [9]. Children were recruited shortly after birth between 1999 and 2000 in Gateshead, an urban district in northeast England. All infants born to mothers resident in Gateshead in pre-specified weeks were eligible for recruitment. The study recruited 1029 infants, 82% of those eligible. The study sample comprised largely (98%) of white British children and was in most respects representative of the north of England. Details of recruitment and description of data collected since birth have been published elsewhere [10].

### Parental perception

Parents were visited at a convenient time at home where they were asked to complete questionnaires on health and lifestyle related issues including their perception of their child’s weight status and childhood overweight. Parental perception of their child’s weight status was assessed using both quantitative and qualitative techniques [2]. For the purpose of the current analysis, only quantitative techniques will be discussed. In brief, parents’ perception of their child’s weight status was assessed using the question ‘How would you describe your child’s weight at the moment? Child’s weight status was grouped into five categories: very underweight, underweight, normal, overweight, and very overweight [11]. Parental concern over their child’s future weight status was assessed by asking: ‘How concerned are you about your child becoming overweight in the future’. Parents were given five choices: unconcerned, a little concerned, concerned, fairly concerned, and very concerned. Parental perspectives of children’s body weight at a societal level were also examined by asking them to respond with yes, no, or not sure to the following question: ‘Do you feel concerned about the national rise in number of overweight children?’ Parental perception accuracy was assessed by comparing parents’ questionnaire answers against the child’s BMI status as assessed using the International Obesity Task Force (IOTF) BMI classification [12,13]. Parents whose perception did not match their child’s actual BMI classification were deemed to have incorrectly perceived their child’s weight status.

### Dietary assessment

Diet was assessed using the FAST (*Food Assessment in Schools Tool)* food diary method, a method validated for use in young children [14]. The FAST diary combines elements from the food diary and food frequency methods and uses age- and sex-specific portion sizes, derived from the National Diet and Nutrition Surveys (NDNS) [15,16]. Each diary day is divided into six time-slots containing tick-lists of foods commonly consumed by children in the UK, with data being collected prospectively [15,16]. Parents, care-givers, lay-observers, and researchers can easily complete the diary by marking food and drinks consumed on the tick-lists in each time slot [17].

Parents and care-givers were asked to complete the diary over a consecutive four-day period outside school times, while lay-observers and researchers filled in the diaries during school time, including time spent at breakfast clubs and after school clubs. The diaries included two weekdays and two weekend days. Full written instructions on how to complete the diary were provided to parents. Dietary data were coded using McCance and Widdowson’s food tables [18].

### Anthropometry, maternal education and economic status

Child and maternal height was measured by trained researchers to nearest 0.1cm using a Leicester portable height measure with the head positioned in the Frankfort plane. Weight was recorded to the nearest 0.1kg using Tanita scales TBF-300MA (TANITA Corporation, Tokyo, Japan). BMI was calculated as weight in kilograms divided by the square of height in meters. Mother’s BMI was classified according to the World Health Organisation (WHO) BMI cut-offs as normal weight, overweight, or obese [19]. In children, BMI was categorised using IOTF cut-offs as underweight, normal weight, overweight and obese [12].

Maternal education was assessed as the mother’s highest educational qualification at time of child’s birth. Household economic status was estimated on the basis of employment status, home and car ownership, with higher status families having at least one wage earner in the household, and owning their home and car.

### Statistical analysis

Foods and beverages were categorised into 38 food groups based on the categories available in the FAST diary. Dietary patterns were derived using principal components analysis (PCA), with food groups entered as grams of intake. PCA is a statistical method used to convert a set of related variables into a smaller set of linearly uncorrelated variables, known as principal components [20]. Principal components are used to describe the variance within a dataset [20]. Varimax rotation was used to derive optimal uncorrelated food patterns and to increase the representation of food groups in each dietary pattern. To decide the number of dietary patterns to retain, a scree plot of eigen values was plotted. Four dietary patterns were selected out of the 15 dietary patterns with eigen values above one. These dietary patterns were mutually independent with a mean of 0 and an SD of 1. Dietary patterns were named based on foods with loading above or below 0.2. For each of the dietary patterns, a score was assigned for each child by summing up the observed consumption of food groups weighted by the factor loadings. A higher score indicates greater conformity with the specific dietary pattern. Regression analysis was used to assess the relationship between accuracy of parental perception of child’s weight status and each of the four dietary patterns, before and after adjustment for potential confounders such as sex, child’s BMI, mother’s BMI status, maternal qualification and economic status. Differences in food group intake, sex, child’s and maternal BMI status, maternal education and family economic status between parents who correctly perceived their child’s weight status and those who incorrectly perceived their child’s weight status were compared using Mann-Whitney for continuous variable’s and chi-square for categorical variables.

All analyses were performed using STATA 12. To account for multiple testing, a p-value of ≤0.01 was deemed significant for all tests

## Results

### Missing data

There were 15 sets of twins. One twin from each pair was randomly excluded from analyses. Additionally, preterm children (n = 57) and families of Muslim or Orthodox Judaism religion (n = 39) were excluded from analyses because their diet and growth trajectories differ from the rest of the children. Around 553 parents had data on accuracy of parental perception, of which 361 had complete data on diet and potential confounders. There were no significant differences in child’s sex, child’s IOTF classification, maternal BMI or parental concern over national overweight between those with or without data on accuracy of parental perception of child weight status. Those with missing data on accuracy of parental perception of child weight status were less likely to have a higher degree or equivalent (66.7% vs. 33%; P-value <0.001) compared to those with complete data, and more likely to be from a more deprived background (53.2% vs. 46.8%; P-value <0.001).

### Parental perception

Of the 361 parents who provided complete data on confounders and on their perception of their child’s weight status, 63 (17%) parents perceived their child as being of ‘normal’ weight when they were actually ‘overweight’ or ‘obese’, or ‘overweight’ when they were actually ‘obese’. No parent overestimated their child’s weight status. Parents who did not correctly perceive their child’s weight status were more likely to have an overweight or obese child than a child with a healthy weight child (100.0% vs. 6.4%; P-value <0.001). They were also more likely to be overweight or obese than to be of a healthy weight (68.3% vs. 45.0%; P-value = 0.002) (Table 1). Parents who did not correctly perceive their child’s weight status were more likely to be concerned over future child overweight (P-value <0.001) but not over the national rise in overweight and obesity (P-value = 0.126) (Table 1).

**Table 1:** Characteristics of study population (n = 361). Data are presented as counts and percentages.

		Parental perception status				
		Correct perception (n = 298)		Incorrect perception (n = 63)		
		n	%	n	%	P-value
Child's gender	Boys	142	(47.7)	32	(50.8)	0.650
	Girls	156	(52.3)	31	(49.2)	
Child's IOTF classification*	Healthy weight	279	(93.6)	0	(0)	**<0.001**
	Overweight	15	(5)	48	(76.2)	
	Obese	4	(1.3)	15	(23.8)	
Parental perception of child's weight status	Normal	286	(96)	54	(85.7)	**0.003**
	Overweight	11	(3.7)	9	(14.3)	
	Very overweight	1	(0.3)	0	(0)	
Mother's BMI classification	Healthy	164	(55)	20	(31.7)	**0.002**
	Overweight	81	(27.2)	22	(34.9)	
Concern over future child overweight	Obese	53	(17.8)	21	(33.3)	
	Unconcerned	182	(61.1)	18	(28.6)	**<0.001**
	A little concerned	84	(28.2)	27	(42.9)	
	Concerned	8	(2.7)	5	(7.9)	
	Fairly concerned	15	(5)	8	(12.7)	
	Very concerned	9	(3)	5	(7.9)	
Concern for national rise of overweight	Yes	214	(71.8)	53	(84.1)	0.126
	No	20	(6.7)	2	(3.2)	
	Not sure	64	(21.5)	8	(12.7)	
Maternal qualification	Degree or equivalent	73	(24.5)	12	(19)	0.823
	A levels or equivalent	29	(9.7)	6	(9.5)	
	GCSEs or equivalent	152	(51)	35	(55.6)	
	NVQs or none	44	(14.8)	10	(15.9)	
Economic Status—based on employment status, home and car ownership	Higher	109	(36.6)	25	(39.7)	0.643
	Lower	189	(63.4)	38	(60.3)	

* Abbreviations: International Obesity Task Force (IOTF), Body Mass Index (BMI), General Certificate of Secondary Education (GCSE), National Vocational Qualifications (NVQ)

### Dietary patterns

Overall, a higher proportion of children whose parents misperceived their weight status reported consuming reduced sugar carbonated drinks (52.4% vs. 33.6%; P-value = 0.005) (Table 2). There was a tendency for a higher proportion of children whose parents misperceived their weight status to consume semi-skimmed milk, although this relationship did not reach statistical significance (Table 2). Similarly, there was a tendency for a lower proportion of children whose parents misperceived their weight status to consume wholegrain cereals not high in sugars (Table 2).

**Table 2:** Proportion of consumers within each food group according to parental perception status. Data are presented as counts and percentages.

	Parental perception status				
	Correct perception (n = 298)		Incorrect perception (n = 63)		P-value
	n	%	n	%	
Beans and pulses	130	(43.6)	19	(30.2)	0.049
Biscuits	270	(90.6)	58	(92.1)	0.715
Breakfast cereals refined high sugar	160	(53.7)	34	(54)	0.968
Breakfast cereals refined low sugar	87	(29.2)	19	(30.2)	0.879
Carbonated drink full sugar	135	(45.3)	34	(54)	0.210
Carbonated drink reduced sugar	100	(33.6)	33	(52.4)	**0.005**
Cereal wholegrain high sugar	59	(19.8)	6	(9.5)	0.054
Cereal wholegrain not high sugar	131	(44)	18	(28.6)	**0.024**
Cheese	190	(63.8)	39	(61.9)	0.781
Chocolate and milkshake powder	69	(23.2)	13	(20.6)	0.665
Confectionery, cakes and puddings	295	(99)	62	(98.4)	0.689
Cordial or squash full sugar	165	(55.4)	33	(52.4)	0.665
Cordial or squash reduced sugar	210	(70.5)	48	(76.2)	0.361
Crisps and savoury snacks	246	(82.6)	55	(87.3)	0.357
Eggs	78	(26.2)	14	(22.2)	0.513
Fried chips	141	(47.3)	25	(39.7)	0.269
Fruit	281	(94.3)	58	(92.1)	0.501
Fruit juice	208	(69.8)	40	(63.5)	0.327
Meat, fish, cheese or eggs dishes	130	(43.6)	26	(41.3)	0.732
Milk based puddings	42	(14.1)	13	(20.6)	0.189
Miscellaneous	288	(96.6)	59	(93.7)	0.164
Non-processed meat and fish products	274	(91.9)	59	(93.7)	0.646
Oven chips	157	(52.7)	30	(47.6)	0.465
Paste, rice and dishes	216	(72.5)	51	(81)	0.264
Pizza	159	(53.4)	25	(39.7)	0.049
Potatoes	265	(88.9)	53	(84.1)	0.285
Processed meat and fish products	272	(91.3)	62	(98.4)	0.050
Semi-skimmed milk	206	(69.1)	52	(82.5)	**0.032**
Spreading fats	274	(91.9)	62	(98.4)	0.066
Sugar	58	(19.5)	16	(25.4)	0.289
Supplements	13	(4.4)	2	(3.2)	0.668
Tea or coffee	73	(24.5)	20	(31.7)	0.232
Vegetables	273	(91.6)	55	(87.3)	0.281
Water	264	(88.6)	54	(85.7)	0.522
White bread	279	(93.6)	60	(95.2)	0.627
Wholemeal bread	159	(53.4)	36	(57.1)	0.584
Whole milk	170	(57)	33	(52.4)	0.498
Yogurts	231	(77.5)	48	(76.2)	0.819

**Fig 1:**
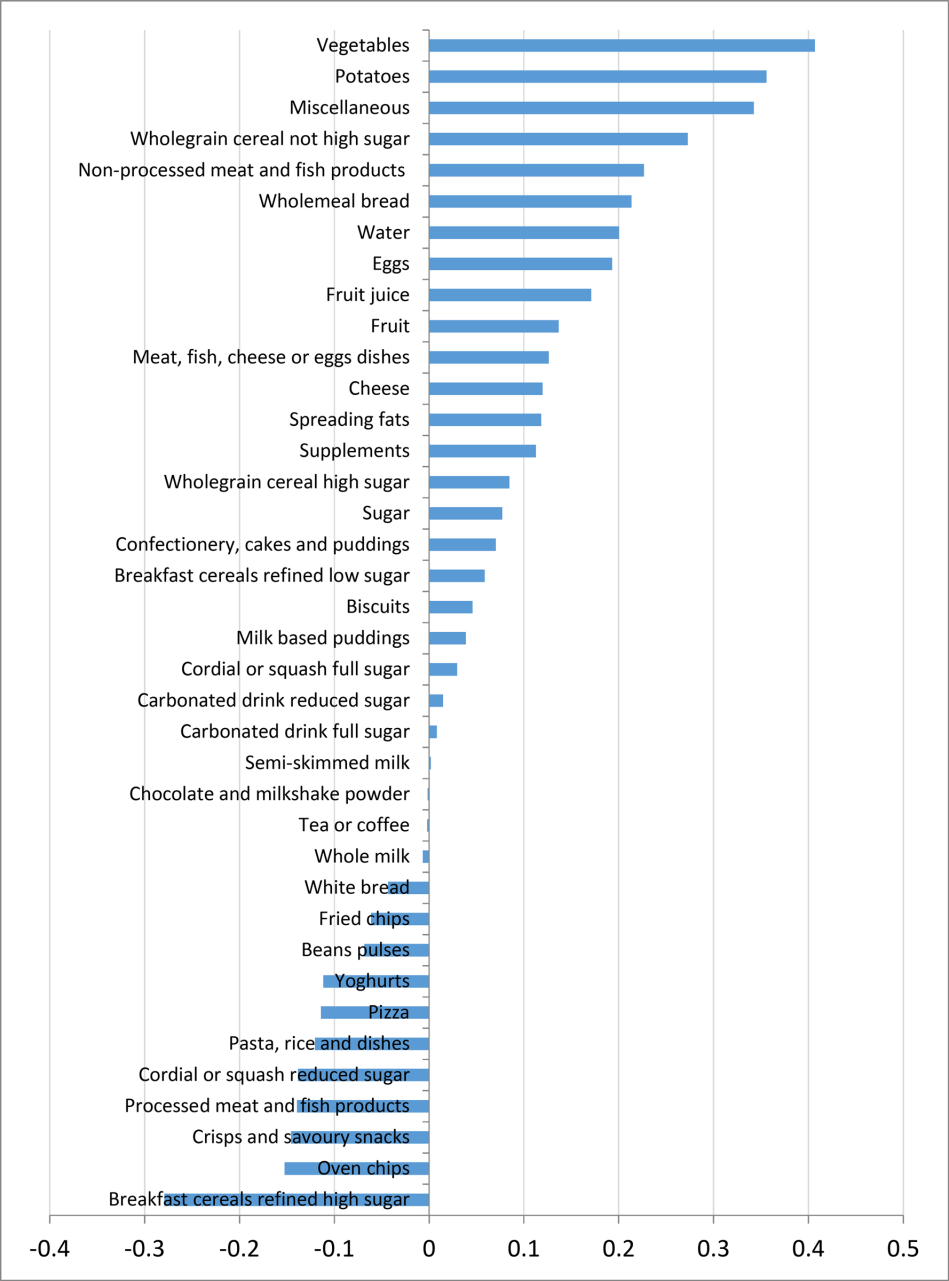
Food group loading of the ‘healthy’ dietary pattern.

Four dietary patterns were derived using PCA accounting for 22.5% of variance of food intake. The first dietary pattern accounted for the largest proportion of variation in food intake (6.7%). This dietary pattern resembled a ‘healthy’ dietary pattern and had positive correlations with vegetables and whole-grain cereal intake, and negative correlations with refined cereals and chips (see Fig 1). The remaining three dietary patterns explained only a small proportion of the variance in food intake and were less interpretable (Figs A-C in S1 File).

After adjustment for confounders, parents who did not correctly perceive their child’s weight had children with a lower ‘healthy’ dietary pattern score compared to children whose parents correctly perceived their weight (β = -0.88; 95% CI: -1.7, -0.1; P-value = 0.028) (Table 3).

**Table 3:** Regression coefficients of the association between parental perception status and other socio-demographic predictors, and the ‘healthy’ dietary pattern score.

		β	95% CI	P-value
Intercept		0.49	(0.0,1.0)	0.064
Perception status	Incorrect perception	-0.88	(-1.7,-0.1)	0.028
Child's sex	Boys	Reference		
	Girls	0.11	(-0.2,0.4)	0.494
Child's IOTF* 8yrs	Normal weight	Reference		
	Overweight	0.85	(0.1,1.6)	0.023
	Obese	0.47	(-0.5,1.4)	0.325
Maternal BMI* status 8yrs	Normal weight			
	Overweight	-0.06	(-0.4,0.3)	0.747
	Obese	-0.17	(-0.6,0.3)	0.431
Maternal qualifications	Degree or equivalent	Reference		
	A levels or equivalent	0.09	(-0.5,0.7)	0.773
	GCSEs or equivalent	-0.90	(-1.3,-0.5)	<0.001
	NVQs or none	-1.23	(-1.8,-0.6)	<0.001
Economic Status—based on employment status, home and car ownership	Higher	Reference		
	Lower	0.20	(-0.2,0.6)	0.296

* Abbreviations: International Obesity Task Force (IOTF), Body Mass Index (BMI), General Certificate of Secondary Education (GCSE), National Vocational Qualifications (NVQ)

## Discussion

### Summary of main findings

The present study aimed to examine the association between accuracy of parental perception of child weight status and children’s dietary patterns. Four dietary patterns were identified which together accounted for 22.5% of total variance in food consumption. The first dietary pattern was the most interpretable and explained the largest proportion of the variance in food intake. This dietary pattern correlated positively with intake of vegetables, potatoes, wholegrain cereals not high in sugars and non-processed meat and fish, and negatively with intakes of refined higher sugar breakfast cereals, chips, crisps and processed meat and fish products. Overall, children whose parents misperceived their weight were found to score lower on this ‘healthy’ dietary pattern compared to children whose parents correctly perceived their child’s weight status.

To our knowledge, this is the first study to characterise dietary patterns based on accuracy of parental perception of child weight status. Only one previous study has investigated the relationship between perception of weight status and food intake [21]. In the latter study, adolescents who underestimated their weight status were more likely to have an unhealthy diet characterised by higher daily consumption of fast food and unhealthy snacks [21]. However, this study differed from our study in that it focused on adolescents’ perceptions of their own weight status as opposed to parents’ perception’ of their child’s weight status. It also assessed individual food groups as opposed to deriving patterns of the whole diet.

Accurate parental perception of weight status is known to be related to lower weight gain over time [22]. There is also evidence to suggest that parents who incorrectly perceive their child’s weight status are less likely to be concerned about their child’s weight and hence engage in behaviour that will encourage weight loss or improve lifestyle factors [4,5]. Our findings are partially in agreement with previous research [5] as they demonstrate that overweight or obese parents more often misperceived their child’s weight status as being normal when their child was actually overweight or obese. Our study also adds to current research by demonstrating that children whose parents misperceived their weight status have overall an unhealthier diet compared to children whose parents correctly perceived their weight. This occurred despite the higher proportion of consumers of diet products, such as reduced sugar carbonated drinks and to a lesser extent semi-skimmed milk, within the former group of children.

Several explanations could be applied to our findings. For instance, it could be speculated that parents with incorrect perception may focus on one aspect of the diet, such as restricting sugar intake, as opposed to the overall diet. Indeed, one recent study reported that that the majority of parents claimed that they enforced healthy eating habits by limiting intake of ‘junk food’ and sweetened drinks and juices [23]. In the latter study, most parents were also found to underestimate their child’s weight status [23]. This disconnect between parental perception of their child’s weight status and diet and child’s actual weight status and dietary habits could be attributed to maternal education. Previous research has demonstrated the importance of maternal education as a determinant of food choices and overweight and obesity [23,24]. In the present study, a significant association between maternal education and the ‘healthy’ dietary pattern was also observed. Mothers with lower educational levels had children with lower ‘healthy’ dietary pattern scores compared to mothers with higher educational levels. Together these findings may point towards the importance of improving parental knowledge of nutrition and the need to educate parents on the different aspects of healthy eating.

It is noteworthy that, no association between maternal deprivation and the healthy dietary pattern was observed in our study. Similarly, there were no differences in maternal education or deprivation status according to parental misperception category. This is in contrast to previous research that has found significant differences in maternal education and deprivation between parents who correctly perceived and those who incorrectly perceived their child’s weight status [23,24]. In general, parents with lower educational attainment have been reported to be less likely to perceive their child’s weight status correctly [23,24]. This association between maternal education and perception of weight status has been found to occur independently of parents’ socioeconomic status, implying that maternal education is critical to recognising overweight and obesity in children [25]. To date, it remains unknown as to whether educating parents improves parental perception of child’s weight status [5]. Nevertheless, based on our results and previous research, it could be argued that maternal education is an important determinant of children’s dietary patterns and may, hence, be central to improving children’s diet.

In the current study, parents who misperceived their child’s weight status had higher concern over their child’s future weight status compared to parents who correctly perceived their child’s weight. This finding is in contrast to previous research which found that parents of overweight children were not concerned over their child’s future weight status [4,5]. These differences could be attributed to differences in the definition of incorrect parental perception. For instance, in the current study some parents reported that their child was overweight when they were actually obese. Thus, some parents may have acknowledged their child’s overweight as a problem but failed to distinguish between overweight and obesity. Distinguishing overweight and obesity is important as research suggests that labelling a child’s weight as overweight does not provide sufficient motivation to trigger changes in lifestyles[26]. This finding highlights once again the need to raise awareness of overweight and obesity in children and suggests the need to assist parents in recognising and defining an unhealthy body weight in children. The latter may also explain why the proportion of children who consumed reduced sugar drinks was higher amongst children whose parents underestimated their weight status, as some parents may have attempted to restrict intake of sugary drinks in their obese child but may have nonetheless underestimated their weight status as being overweight. One previous study reported that parents who recognised their adolescent children as being overweight were more likely to encourage their children to diet [22]. The latter is partially consistent with our findings and raises a point of concern as focusing on one aspect of the diet, instead of the whole diet, may not necessarily translate into effective and healthy eating behaviour [22].

Our study reinforces the importance of examining the diet as a whole instead of focusing on individual food groups or nutrients. This is because analysis based on individual food groups showed higher consumption of diet products such as reduced sugar carbonated drinks in children whose parents misperceived their weight status. However, once the correlation between different food groups was taken into account, it was evident that children whose parents misperceived their weight status were less likely to follow a healthy diet. Indeed, the main strength of utilising PCA lies in its ability to combine information across the diet based on food intakes, taking into consideration the complexity of the diet and the fact that foods and/or nutrients are not consumed in isolation [11]. This approach helps unravel underlying food consumption patterns, rendering PCA more pertinent to assessing dietary behaviour and food choices compared to analyses based on individual foods and/or nutrients [11].

### Strengths and Weakness of the Study

There are several limitations to the current analysis. To illustrate, ethnic differences in parental perception were not investigated. Ethnicity has been shown to be an important predictor of parental perception by some studies [27,28] but not others [29]. Ethnic differences in parental misperception may arise as a result of differences in cultural and societal definitions of overweight [28]. As a result, findings from the current studies are limited, as they could not be generalised to other ethnic groups. Moreover, data on parental perception and children’s diet was only analysed at one time point, as it was not possible to consolidate dietary data from other time points due to changes in dietary assessment methods and lack of data on parental perception of child weight status. Thus, further research will be required to examine if the unhealthy dietary patterns in children whose parents misperceived their weight status tracks into adulthood. Additionally, sample size was small which did not permit further stratification of data by overweight and obesity categories. Quantitative data of parental diet was also not available. The latter would have permitted investigation of the impact of parental diet on children’s dietary patterns. Finally, although diet was assessed using a validated food diary method over a period of four days, no data on portion sizes at individual level were collected. This meant that children were assigned pre-specified portion sizes, which may have weakened some of the observed associations. Indeed, the four dietary patterns obtained in the current study accounted for a small variation in food intake and only significant associations between parental misperception and the first dietary pattern were observed. The latter is, nevertheless, consistent with previous studies in children, which found that PCA derives dietary patterns that explain only a small amount of the total variance of diet in the population [11].

Regardless of these limitations, the current study possesses several strengths. For instance, parental perceptions of children’s weight status were assessed in a large population-based birth cohort and data on weight and height were objectively measured. In addition, the study sample was socio-economically representative of families living in the Northeast of England [2,10].

### Conclusion and Implications

In conclusion, parental perception of child’s weight status is an important predictor of children’s diet. Parents who did not correctly perceive their child’s weight status had children who scored lower on the ‘healthy’ dietary pattern, despite higher consumption of ‘diet products’ such as reduced sugar carbonated drinks. These findings suggest parental perception as an important target for public health interventions. Moreover, our study carries important implications to tackling the current obesity epidemic since previous evidence suggests that parents who fail to acknowledge the overweight and obesity problem are unlikely to respond to guidance provided by health care professionals or public health policies. Future research should focus on defining how parental perception influences parents’ diets and to examine if a child’s or parent’s diet mediates the association between parental perception and child weight. Furthermore, further work is required to identify and improve current methods of assessing parental perception of children’s weight status. Finally, considering that 77% of overweight children become obese adults [30], additional research is needed to investigate the long-term consequences on health of overweight and obese children whose parents misperceived their weight status.

## Supporting Information

S1 Dataset Dataset of variables used in analysis. (XLS)

S1 File Food group loading for various dietary patterns. Fig A. Food group loading of dietary pattern 2. Fig B.Food group loading of dietary pattern 2.Fig C. Food group loading of dietary pattern 2.
(DOCX)

## Abbreviations

BMI: Body Mass Index
FAST: *Food Assessment in Schools Tool*
GCSE: General Certificate of Secondary Education
GMS: Gateshead Millennium Study
IOTF: International Obesity Task Force
NDNS: National Diet and Nutrition Surveys
PCA: Principal Component Analysis
NVQ: National Vocational Qualifications
WHO: World Health Organisation
