# Supplementary material for: Parental Perception of Weight Status: Influence on Children’s Diet in the Gateshead Millennium Study
Source: PLoS One. 2016 Feb 17;11(2):e0144931. doi: 10.1371/journal.pone.0144931 (PMC4757535; doi:10.1371/journal.pone.0144931)
Supplement: S1 File — Fig A. Food group loading of dietary pattern 2. Fig B.Food group loading of dietary pattern 2.Fig C. Food group loading of dietary pattern 2. (DOCX) [file pone.0144931.s002.docx]

Figure A Food group loading of dietary pattern 2.

Figure B Food group loading of dietary pattern 3.

Figure C Food group loading of dietary pattern 4.
